# From Cardiovascular Prevention and Differential Diagnosis to the Treatment of Myocardial Damage—Experimental and Human Perspectives

**DOI:** 10.3390/ijms242316997

**Published:** 2023-11-30

**Authors:** Jan Pitha

**Affiliations:** Department of Cardiology, Laboratory for Atherosclerosis Research, Institute for Clinical and Experimental Medicine, 140 21 Prague, Czech Republic; japi@ikem.cz

Cardiovascular diseases are characterized by many clinical, morphological, functional, and biochemical markers, including age, sex, genetic factors, plasma lipids, glycemia, and many other laboratory parameters. These parameters can be used to predict the prognosis of patients with these diseases. The most frequently discussed of which is coronary heart disease, which includes acute coronary syndrome and myocardial infarction [1]. This Special Issue entitled “Mechanisms of Cardiovascular Disease: Molecular Perspective 2.0” of the *International Journal of Molecular Sciences* includes six contributions: five original articles and one review. All of these papers present studies that mainly focus on molecular aspects of cardiovascular disease, including interesting intervention studies in experimental models. In general, cardiovascular diseases cover a wide spectrum of disorders, affecting several functions from myocardium to microcirculation. The interplay between myocardial tissue, central, peripheral vessels, and microcirculation could be used to define patients at risk, and knowledge of the mechanisms behind these processes could improve the fate of such patients. Articles published in this Special Issue focus on several areas of research on cardiovascular diseases, including prevention, treatment and deeper insights into some of their mechanisms. As in all such studies there always remain some limitations regarding the immediate impact of such findings on clinical practice. However, the results of these studies might represent the first steps towards clinical implications or, at the very least, new directions of future research.

Amra Jujic and co-authors [2] presented a combined set of data from human and experimental studies focusing on plasma galectin-4 (Gal-4), a protein found in the gastrointestinal tract. This protein supposedly plays a role in the apical trafficking of proteins that are important for development of cardiovascular diseases, and it could be especially crucial for the treatment of diabetic patients. Gal-4 is interconnected with other metabolic factors responsible for the effects of incretin, which could be defective in individuals with metabolic diseases such as diabetes mellitus. The imbalance of this system might lead to vascular adverse effects. On the contrary, evidence suggests that incretin-based antihyperglycemic agents, including dipeptidyl peptidase-4 and glucagon-like peptide 1 receptor agonists, exert beneficial effects in diabetic patients and could be targets for the prevention and treatment of vascular diseases such as ischemic strokes. In an observational study of approximately 1700 participants (Malmö Preventive Project), plasma Gal-4 was found to be higher in subjects with ischemic stroke with an odds ratio adjusted for age, sex, and other metabolic variables of 1.52 (95% confidence interval 1.01–2.30). In the second experimental part of the study, plasma Gal-4 increased after experimental stroke in mice fed a high-fat diet, but also in mice with a control diet. On the one hand, an observational study in patients following stroke is not the strongest evidence for causal relationship between a given factor and cerebrovascular disease. On the other hand, when such evidence is supported by an experimental study, this could indicate potential importance of this factor. As the authors admit, the relationship between Gal-4 and stroke should be confirmed in future physiological and clinical studies before it can be labelled as a valuable prognostic marker for stroke and its outcomes; further evidence is required to study it as the therapeutic target. Despite stroke being such a debilitating condition, it certainly deserves to be investigated from all aspects, including the study of metabolic biomarkers such as plasma galectin-4.

Regarding preventive measures, particular pathophysiological mechanism of the protective effect of exercise on cardiovascular system was proposed by Mi Zhang and colleagues [3] on the basis of data from their experimental study. In a well-designed and very sophisticated study, the authors focused on the pathophysiology of changes induced by exercise in a mice model. In particular, they tested the resistance of isoprenaline to sympathetic activation, including the production of reactive oxygen species and other potentially deleterious factors. They found that exercise training attenuated the production of reactive oxygen species, but also weakened the activation of a gene from the NLR family, the pyrin domain-containing 3 (NLRP3) inflammasome, in an AMPK-dependent manner, meaning that its proinflammatory status was diminished. In addition, they found that exercise training alleviated not only cardiac macrophage infiltration induced by isoprenaline, but also chemokines and the expression of proinflammatory cytokines in wild-type mice. They studied these mechanisms on a molecular level, and based on their results, they also proposed that the effects of isoprenaline were inhibited in cardiomyocytes by metformin (MP-activated protein kinase (AMPK) activator) pretreatment. Exercise is frequently recommended for most people with metabolic and cardiovascular health problems. Nevertheless, the exact pathophysiological mechanisms of this lifestyle measure are far from being completely understood. Although this study presents solely experimental data, a deeper understanding of the mechanisms of exercise could lead us to implement treatment approaches for our patients and clients with more confidence.

In their pilot experimental study, David Schumacher et al. [4] focused on the modulation inside myocardial tissue, especially regarding changes in extracellular matrix collagen after myocardial infarction. In particular, they studied the expression of collagen subtypes and the role of isolated immune cells in wild and knockout mouse models (C57BL/6 wild-type mice, as well as CCR1−/− mice) after myocardial infarction. In an extensive methodological study featuring a wide range of measurements that include primers to determine the mRNA expression of collagen species and associated connective tissue factors, the researchers proposed that immune system, particularly immune cells activated after myocardial infarction, can substantially control collagen synthesis and deposition. Despite the fact that this is a pilot study based on a low number of experimental models (*n* = 3–5), the process of myocardium healing, and particularly the role of connective tissue, could also be significant due to the subsequent development of heart failure, including heart failure with a preserved ejection fraction. The latter could be associated with changes in myocardial connective tissues caused by acute and chronic ischemia. Therefore, although the presented results are far from finalized, they could be source of inspiration for further studies focusing on myocardial dysfunction caused by the interplay between immune system and connective tissue in ischemic heart disease.

In an elegant experimental study, Yichen Xu et al. [5] focused on the topic of connective tissue changes after myocardial infarction, similar to the study by Schumacher et al. [4]; however, they went further and investigated the effect of vitamin C at various doses in a mice model. Concentrations from 0 to 50 ug/mL (0, 10, 20, 30, 40, 50 ug/mL) were used. The main goal was to establish the effect of vitamin C on the primary function of cardiac fibroblasts in response to collagen synthesis. The researchers presented evidence that vitamin C at an optimal dose (10 ug/mL) could restore equilibrium in the process of healing after myocardial infarction. They proposed that vitamin C at this concentration is able to favorably reprogram the respiratory mitochondrial metabolism and prevent the increased accumulation of intracellular reactive oxygen species. In addition, the collagen substrate potentiated the modulator role of vitamin C in reinforcing the structure of type I and type III collagen synthesis by efficiently reducing the expression of collagen V. Favorable data were also observed regarding the synergistic function of vitamin C at an optimal dose in maintaining the balance of radical scavenger and gene transcription, which is important in the initial phases of healing after myocardial infarction. On the one hand, they found that vitamin C at optimal dose reduces high levels of antioxidants, leading to increased mitochondrial respiration. On the other hand, the cytotoxic effects of vitamin C at high doses on mitochondrial activity and impaired collagen synthesis were also detected. The role of vitamins is still intensively discussed, and cautious approaches to their supplementation (including vitamin C) are being proposed for humans. From the perspective of future research, the main takeaway from this study is that the dose and timing of any treatment, not only with vitamin C but in general, could be essential.

Xavier Poirot-Seynaeve et al. [6] studied the potential role of procalcitonin (PCT) in inflammatory disorders of the vasculature, namely ANCA-associated vasculitis (AAV). For their analysis, they used retrospective data from patients with established AAV. They conclude that PCT might be useful in the differential diagnosis in AAV, particularly for differentiating between the activation (flare) of vasculitis and a “regular” infection. These findings are based on a retrospective study of 74 patients with 3 different types of vasculitis. They observed that PCT was significantly higher in the group with infection than in the group with a flare of vasculitis with 53% sensitivity and 74% specificity (threshold of 0.2 µg/L). The level of C-reactive protein (CRPs) was also significantly higher in cases of infection compared to the relapse of AAV; its sensitivity was 94.2% and specificity was 11.3%. Fibrinogen, white blood cell count, eosinophil count, and neutrophil count did not significantly differ between the studied groups. In the multivariate analysis, the relative risk of infection was 2 [1.02; 4.5] for a PCT above 0.2 µg/L. The authors conclude that, in AAV, PCT may be useful for discriminating between infections and a flare of vasculitis in patients suffering from AAVs. The authors discuss the future use of other markers, such as presepsin and endocan, but their study is especially valuable since they investigated markers that are widely available in general practice. Because of the relatively low number of selected patients, the question remains regarding how these results could be generalized for general practice. In addition, other factors, regularly available in clinical practice, could be used for a differential diagnosis, including systemic inflammation response system [7]. Despite their limitations, these factors provide data that could help in everyday clinical practice and care for patients with vasculitis using easily available parameters.

Finally, Liam S. Couch et al. [8] presented an overview of Takotsubo syndrome, mainly focusing on molecular mechanisms, including a wide range of biomarkers for this ill-defined entity. An early breakthrough in the knowledge of pathophysiological mechanisms is not expected in this field; reliable data are not available from original studies, yet it is important to collect and present such data where possible. In this case, a well prepared overview is presented, which includes hormonal, hemodynamic, genetic, and other factors associated with Takotsubo syndrome, maintaining the principle that catecholamine surge is its primary cause. This review might be expanded by a more extensive discussion that focuses on sex hormones and the widely known process of estradiol depletion, the latter of which covers the (lack of) role of the testosterone in women. Some authors even propose the term Taktsubo endocrinopathy [9,10]. More speculative insights into treatment possibilities at least at a molecular/experimental level could also be discussed.

In conclusion, in this Special Issue of *IJMS*, several studies were presented that focus on the prevention and treatment of vascular diseases, mainly at the level of molecular mechanisms and/or the spectra of biomarkers used for the identification of treatment targets. In particular, two papers focused on remodeling myocardial tissue after myocardial infarction in experimental models. Moreover, one pilot study and a more extensive study mainly focused on the effects of vitamin C at different doses and multiple subcellular levels. Another experimental study offered newer and more detailed pathophysiological insights into the effect of exercise on the cardiovascular system. Another study focused on metabolic biomarkers, one of which could be used for the prediction of stroke in humans, namely, galectin-4 and supported this finding also by an experimental data. Another study focused on the challenges for regular physicians and proposed an improvement in the differential diagnosis for the activation of ANCA-associated vasculitis. Additionally, an overview of Takotsubo syndrome provided valuable information regarding recent evidence about this entity.
